# Cu_3_(PO_4_)_2_: Novel Anion Convertor for Aqueous Dual-Ion Battery

**DOI:** 10.1007/s40820-020-00576-1

**Published:** 2021-01-04

**Authors:** Haoxiang Yu, Chenchen Deng, Huihui Yan, Maoting Xia, Xikun Zhang, Zhen-Bo Wang, Jie Shu

**Affiliations:** 1 School of Materials Science and Chemical Engineering, Ningbo University, Ningbo, 315211 Zhejiang People’s Republic of China; 2 MIIT Key Laboratory of Critical Materials Technology for New Energy Conversion and Storage, School of Chemistry and Chemical Engineering, State Key Lab of Urban Water Resource and Environment, Harbin Institute of Technology, Harbin, 150001 Heilongjiang People’s Republic of China; 3 Key Laboratory of Advanced Energy Materials Chemistry (Ministry of Education), College of Chemistry, Nankai University, Tianjin, 300071 People’s Republic of China

**Keywords:** Dual-ion battery, Aqueous electrolyte, Cu_3_(PO_4_)_2_, Electrochemistry, Three-electrode cell

## Abstract

- A novel anion electrode Cu_3_(PO_4_)_2_ is proposed at the first time.
- The reaction mechanism of Cu_3_(PO_4_)_2_ electrode is investigated.
- The dual-ion cell is constructed by using pretreated Cu_3_(PO_4_)_2_ and Na_0.44_MnO_2_.

**Supplementary Information:**

The online version of this article (10.1007/s40820-020-00576-1) contains supplementary material, which is available to authorized users.

## Graphical abstract

Electrode materials which can reversibly react with anions are of interest for aqueous dual-ion batteries. Herein, we propose a novel anion electrode, Cu_3_(PO_4_)_2_, for constructing an aqueous dual-ion cell. The Cu_3_(PO_4_)_2_ electrode can operate in a quasi-neutral condition and deliver a reversible capacity of 115.6 mAh g^−1^ with a well-defined plateau at −0.17 V versus Ag/AgCl. Its reaction mechanism shows that Cu_3_(PO_4_)_2_ decomposes into Cu_2_O and subsequently is converted into Cu during the initial discharge process. In the following charge process, Cu is oxidized into Cu_2_O. It suggests Cu_3_(PO_4_)_2_ reacts with OH^−^ ions instead of PO_4_^3−^ ions after the initial discharge process and its potential thereby depends upon the OH^−^ ions concentration in electrolyte. Additionally, an aqueous dual-ion cell is built by using pretreated Cu_3_(PO_4_)_2_ and Na_0.44_MnO_2_ as anode and cathode, respectively. During cycling, OH^−^ ions and Na^+^ ions in electrolyte can be stored and released. Such a cell can provide a discharge capacity of 52.6 mAh g^−1^ with plateaus at 0.70 and 0.45 V, exhibiting the potential of application. This work presents an available aqueous dual-ion cell and provides new insights into renewable energy storage and adjustment of the OH^−^ ions concentration in aqueous buffer solution.

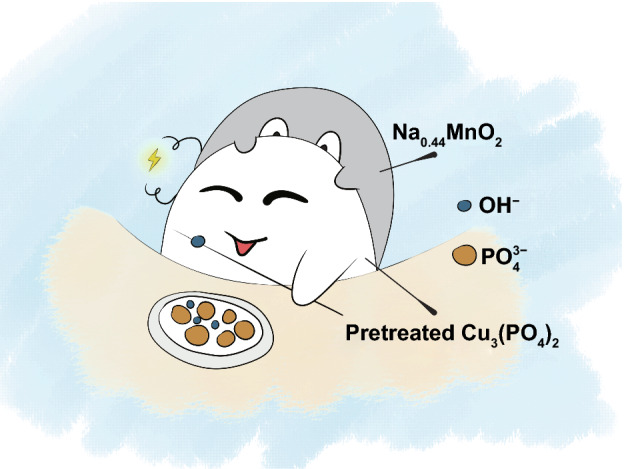

## Introduction

For the storage of energy coming from renewables such as solar and wind, numerous efforts have been dedicated to the development of rechargeable battery over past several decades [1, 2]. Among the multitudinous explored rechargeable batteries, aqueous dual-ion battery as the novel energy storage device has attracted intensive attention recently because of its availability, low cost, high safety and eco-friendliness [3–6]. Its concept is different from that of tradition rocking-chair battery in which anions or cations migrate across electrolyte and then react with anode and cathode [7, 8]. For aqueous dual-ion battery, anions react reversibly with the electrode, whereas cations do the same way in the other electrode. It is developed from dual-carbon batteries or dual-graphite batteries as scientists find that anions can be inserted into graphite [9, 10]. The first prototype of dual-ion batteries used nonaqueous electrolytes and carbonaceous electrodes are proposed by McCullough et al. [11]. In that patent, the electrochemical behavior of this battery is described according to the “dual-intercalation” mechanism. Thereafter, continuous progress is made to the development of dual-ion batteries [12–14]. Although traditional dual-ion batteries using organic electrolytes (including ionic liquid electrolytes) exhibit high safety, high working voltages (normally > 3 V), and reasonable specific capacity (~ 80 mAh g^−1^), the flammability and toxicity of organic electrolytes make them suffer from the safety issues [13, 15–22]. These problems hinder their wide application. To solve these problems, dual-ion batteries with nonflammable and low toxicity aqueous electrolytes have been proposed, and several configurations such as Ag/MnO_2_ [23], NaTi_2_(PO_4_)_3_/Bi [24], and NaTi_2_(PO_4_)_3_/Ag [25, 26] have been demonstrated and fabricated so far. Notably, these reported systems use silver (Ag) and bismuth (Bi) as the electrodes to capture the anions. Although the performance of these materials shows decent, they possess several drawbacks which need to be conquered. Ag is a little bit expensive in price, whereas Bi can hardly react with anions in a mild solution. Thus, constructing an available aqueous dual-ion battery which can cycle in a quasi-neutral condition is of the great importance and desired.

The update of aqueous dual-ion battery depends on the selection of electrode materials which acts as its key components. Many literatures have reported the electrode materials for releasing/storing the cations [27, 28]. Yet studies for investigating the anion containers are relatively less. Hence, we herein demonstrate a novel anion container, Cu_3_(PO_4_)_2_, for constructing an aqueous dual-ion cell. This material can operate in a quasi-neutral condition with well-defined plateaus and good performance, and its price is lower than that of Ag, although its reaction mechanism is far different from our original vision. We also use the pretreated Cu_3_(PO_4_)_2_ as anode to assemble the aqueous dual-ion cell coupled with Na_0.44_MnO_2_ as cathode. It presents well-defined operating plateaus and good cycling performance.

## Results and Discussion

**Fig. 1:**
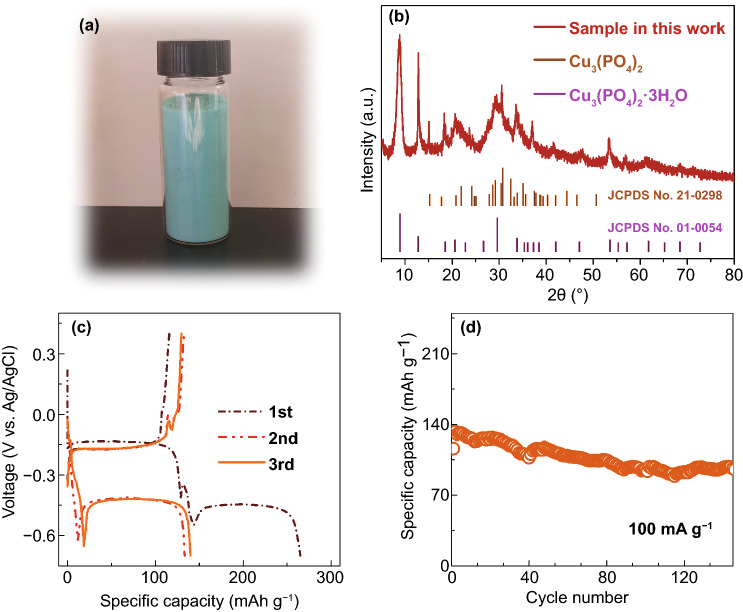
**a** Digital photo and **b** XRD pattern of Cu_3_(PO_4_)_2_. **c** Galvanostatic discharge/charge profiles of Cu_3_(PO_4_)_2_ between −0.7 and 0.4 V versus Ag/AgCl at 100 mA g^−1^, and **d** the corresponding cycling performance

Cu_3_(PO_4_)_2_ is an inorganic compound which is composed of copper cations and phosphate anions. Due to its insolubility in water, Cu_3_(PO_4_)_2_ can be prepared by the facile precipitation method. The typical synthesis is described in supporting information. The as-obtained powder is sky blue material as shown in Fig. 1a. X-ray diffraction (XRD) pattern (Fig. 1b) suggests that two phases exist in this powder, which are Cu_3_(PO_4_)_2_ and Cu_3_(PO_4_)_2_·3H_2_O, respectively, according to the two reference patterns. Besides, most of diffraction peaks are found to show the large full width at half maximum, indicative of its small crystallite size. The scanning electron microscope (SEM) images prove this result. As observed in Fig. S1a, b, the Cu_3_(PO_4_)_2_ powder consists of countless nanosheets with thickness around 25 nm, providing large surface area to contact with the electrolyte. Additionally, the water content in this powder is measured by thermogravimetric (TG) analysis (Fig. S2). About 6% of mass is lost below 200 °C, corresponding to the elimination of the physically absorbed and zeolitic water [29].

Cu_3_(PO_4_)_2_ selected as the electrode material in this work is based on its low thermodynamic solubility product [30]. We think when the Cu_3_(PO_4_)_2_ electrode is discharged, Cu will be produced and subsequently a metal-sparingly soluble salt electrode is constructed until Cu_3_(PO_4_)_2_ is vanished. The half reaction should be as follows:

1 $${\text{Cu}}_{3} ({\text{PO}}_{4} )_{2} + 6{\text{e}}^{ - } \Leftrightarrow 3{\text{Cu}} + 2{\text{PO}}_{4}^{{3{ - }}}$$

Its potential thus can be given by:

2 $$\begin{gathered} \begin{array}{*{20}c} E & { = E_{{{\text{Cu}}^{2 + } /Cu}}^{\theta } + \frac{{\text{RT}}}{2F}\ln \alpha_{{{\text{Cu}}^{2 + } }}^{{}} } \\ \end{array} \hfill \\ \begin{array}{*{20}c} {} & { = (E_{{{\text{Cu}}^{2 + } /Cu}}^{\theta } + \frac{{\text{RT}}}{6F}\ln K_{sp} ) - } \\ \end{array} \frac{RT}{{6F}}\ln \alpha_{{{\text{PO}}_{4}^{3 - } }}^{2} \hfill \\ \begin{array}{*{20}c} {} & { = E_{{{\text{Cu}} /Cu_{3} (PO_{4} )_{2} /PO_{4}^{3 - } }}^{\theta } - \frac{{\text{RT}}}{6F}\ln \alpha_{{{\text{PO}}_{4}^{3 - } }}^{2} } \\ \end{array} \hfill \\ \end{gathered}$$

Finally, the potential of Cu_3_(PO_4_)_2_ electrode is calculated to be −0.22 V versus Ag/AgCl.

To verify the aforementioned half reaction and corresponding potential, the Cu_3_(PO_4_)_2_ electrodes are fabricated and tested in three-electrode cells. Figure 1c, d exhibits the galvanostatic discharge/charge profiles of Cu_3_(PO_4_)_2_ electrode and its corresponding cycling performance. Two distinct plateaus around −0.14 and −0.40 V versus Ag/AgCl is observed upon the initial discharge process, whereas only one plateau at −0.17 V versus Ag/AgCl appears in the recharge process. This case leads to that the initial discharge capacity (265.1 mAh g^−1^) is much higher than the following recharge capacity (115.9 mAh g^−1^). We consider that the large irreversible capacity loss during initial cycle is attributed to the formation of several intermediates as some reported metal oxides [31, 32], which can be reacted with lithium ions in the first discharge process. Additionally, the difference between calculated potential and the experimental one is slight. In the following second and third cycles, the large irreversible capacity losses almost disappear, and the charge capacities of Cu_3_(PO_4_)_2_ electrode reach to 132.6 and 129.9 mAh g^−1^, respectively. The differential dQ/dV plots of Fig. 1c are displayed in Fig. S3. An increase in charge capacity may be owing to the fact that electrolyte does not contact well with Cu_3_(PO_4_)_2_ electrode before cycling. After 45 cycles, the Cu_3_(PO_4_)_2_ electrode can deliver a reversible capacity of 115.6 mAh g^−1^ with 87.2% of its second capacity. Even after 145 cycles, the reversible capacity can still be maintained at 96 mAh g^−1^. These results suggest the good cycling performance. If any defects could be introduced into this active material, the cycling performance may be better [33, 34].

**Fig. 2:**
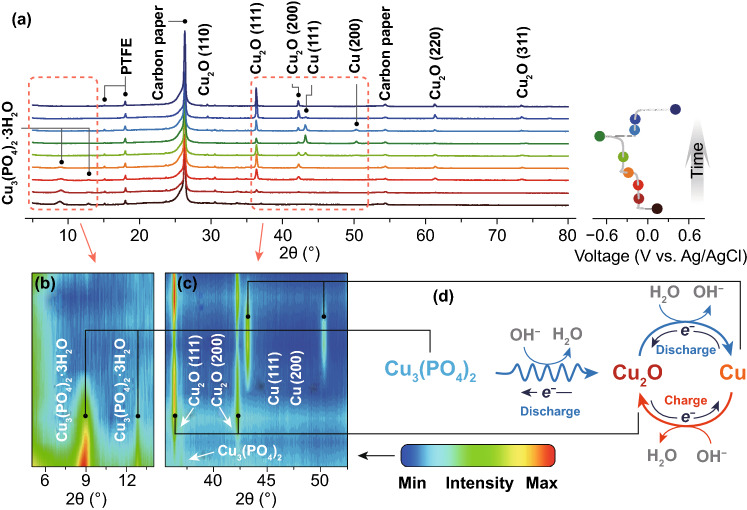
**a** XRD patterns of Cu_3_(PO_4_)_2_ electrode during cycling. The contour map for the corresponding XRD patterns in the 2*θ* range of **b** 5–14° and **c** 35.5–52.5°. **d** Reasonable mechanism for Cu_3_(PO_4_)_2_ electrode during the electrochemical reaction

What are the intermediates during discharging and the corresponding mechanism? To answer these two questions, we have characterized the Cu_3_(PO_4_)_2_ electrodes at various states of discharge by using XRD measurement. The obtained results depicted in Fig. 2 are far different from our original vision. For an as-prepared Cu_3_(PO_4_)_2_ electrode, diffraction peaks belonged to Cu_3a_(PO_4_)_2_ and Cu_3_(PO_4_)_2_·3H_2_O can be defined. Since PTFE binder is electrochemically inactive, we select it as an internal standard to conduct quantitative phase analysis. With discharging to the first plateau at −0.14 V versus Ag/AgCl, the Cu_3_(PO_4_)_2_ and Cu_3_(PO_4_)_2_·3H_2_O diffraction peaks in intensity slowly decrease (Fig. 2b), while two new diffraction peaks assigned to the (111) and (200) facets of Cu_2_O phase appear (Fig. 2c), suggesting that the Cu_3_(PO_4_)_2_ and Cu_3_(PO_4_)_2_·3H_2_O phases slowly decompose into Cu_2_O. During discharging on the second plateau around −0.40 V versus Ag/AgCl, we found that the intensities of the Cu_2_O diffraction peaks in electrode is still decreasing with the formation of Cu phase until the voltage is at −0.7 V versus Ag/AgCl. Thus, the intermediate upon initial discharging is Cu_2_O. In the recharge process, Cu is converted into Cu_2_O instead of Cu_3_(PO_4_)_2_, which is the main reason for the initial irreversible capacity loss. As a result, the electrochemical reaction of the Cu_3_(PO_4_)_2_ electrode during cycling can be described as:

3 $${\text{The first plateau}} :\;2{\text{Cu}}_{3} ({\text{PO}}_{4} )_{2} + 6{\text{e}}^{ - } + 6{\text{OH}}^{ - } \Rightarrow 3{\text{Cu}}_{2} {\text{O}} + 4{\text{PO}}_{4}^{3 - } + 3{\text{H}}_{2} {\text{O}}$$

4 $${\text{The second plateau}} :\;{\text{Cu}}_{2} O + {\text{H}}_{2} O + 2{\text{e}}^{ - } \Rightarrow 2{\text{Cu}} + 2{\text{OH}}^{ - }$$

5 $${\text{The plateau during recharging}} :\;2{\text{Cu}} + 2{\text{OH}}^{ - } \Rightarrow {\text{Cu}}_{2} O + {\text{H}}_{2} {\text{O}} + 2{\text{e}}^{ - }$$

The corresponding mechanism is schematically illustrated in Fig. 2d.

**Fig. 3:**
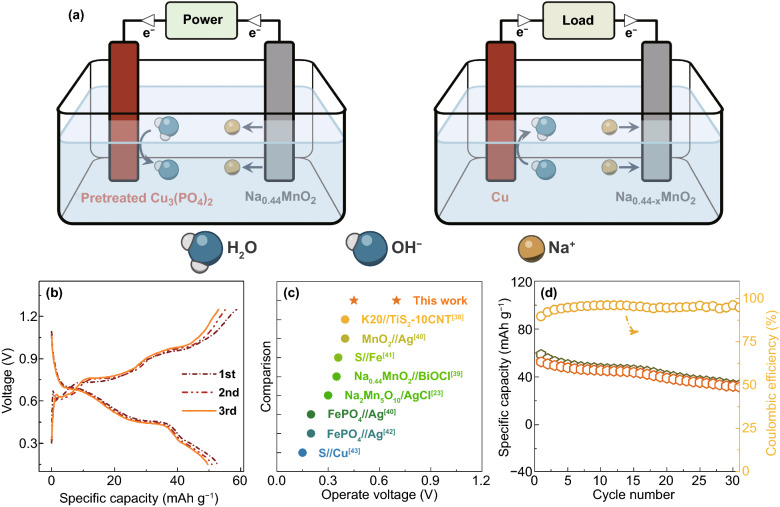
**a** Schematic of pretreated Cu_3_(PO_4_)_2_/Na_0.44_MnO_2_ dual-ion cell for charging and discharging (pretreated Cu_3_(PO_4_)_2_ electrodes is that the Cu_3_(PO_4_)_2_ electrodes is discharged and recharged in half cell for one cycle). **b** Galvanostatic discharge/charge profiles of pretreated Cu_3_(PO_4_)_2_/Na_0.44_MnO_2_ dual-ion cell. **c** Operating voltage of pretreated Cu_3_(PO_4_)_2_/Na_0.44_MnO_2_ dual-ion cell compared to the cells from previous studies. **d** Cycling performance of pretreated Cu_3_(PO_4_)_2_/Na_0.44_MnO_2_ dual-ion cell

According to the electrochemical mechanism mentioned above, the Cu_3_(PO_4_)_2_ electrode reacts with OH^−^ ions instead of PO_4_^3−^ ions after initial discharge process, and its potential depends upon the concentration of OH^−^ ions in electrolyte. The voltage profiles of Cu_3_(PO_4_)_2_ electrodes in electrolytes with different pH validate this result (Fig. S4). It should be noted that although Cu_3_(PO_4_)_2_ electrode provides larger specific capacity and lower plateau in 0.75 M NaH_2_PO_4_ electrolyte and 0.75 M Na_3_PO_4_ electrolyte, respectively, their cycling performances (Fig. S5) are inferior to that of Cu_3_(PO_4_)_2_ electrode in 0.75 M Na_2_HPO_4_ electrolyte (Fig. 1d). Nevertheless, an aqueous dual-ion cell can still be constructed and the corresponding schematic is depicted in Fig. 3a. As viewed, we select Na_0.44_MnO_2_ as cathode due to its low cost and eco-friendliness [35–37]. Its voltage profiles in 0.75 M NaH_2_PO_4_ electrolyte and 0.75 M Na_2_HPO_4_ electrolyte are shown in Figs. S6 and S7, respectively. During charging, Na^+^ ions and OH^−^ ions are released by the Na_0.44_MnO_2_ and pretreated Cu_3_(PO_4_)_2_ electrodes, respectively. Meanwhile, this cell can increase the concentration of NaOH in electrolyte. Upon discharging, these two ions are captured by the cathode and anode, respectively, leading to the reduction in the concentration of NaOH. As a result, this aqueous dual-ion cell can not only modify the concentration of OH^−^ ions in electrolyte, but also provide electrical energy. The reaction of this cell can be written as follows:

6 $$x{\text{Cu}}_{2} O + x{\text{H}}_{2} O + 2{\text{Na}}_{0.44} {\text{MnO}}_{2} \Leftrightarrow 2x{\text{Cu}} + 2x{\text{OH}}^{ - } + 2x{\text{Na}}^{ + } + 2{\text{Na}}_{0.44 - x} {\text{MnO}}_{2}$$

Figure 3b displays galvanostatic discharge/charge profiles of pretreated Cu_3_(PO_4_)_2_/Na_0.44_MnO_2_ dual-ion cell. Due to the presence of irreversible capacity loss in the initial cycle, the Cu_3_(PO_4_)_2_ electrode needs to be pretreated before the dual-ion cell assembly. For the pretreatment, the Cu_3_(PO_4_)_2_ electrode is discharged and subsequently recharged for 1 cycle. As observed in Fig. 3b, this as-fabricated dual-ion cell can provide a discharge capacity of 52.6 mAh g^−1^ at 0.5 C based on the mass of Na_0.44_MnO_2_. Thus, the *x* value in Na_0.44-*x*_MnO_2_ can be calculated, being 0.19. Two well-defined plateaus are observed at 0.70 and 0.45 V. By contrast, recently reported desalination batteries, such as Na_2_Mn_5_O_10_//AgCl [23], TiS_2_//K20 [38], and BiOCl//Na_0.44_MnO_2_ [39], displayed the operating plateaus only at ~ 0.3, ~ 0.4, and ~ 0.1 V, respectively. The detailed comparison for operating voltage of pretreated Cu_3_(PO_4_)_2_/Na_0.44_MnO_2_ dual-ion cell in this work with other cells in the literatures [23, 38–43] is plotted in Fig. 3c and Table S1. It is worth noting that the pH value changes during cycling as shown in Fig. S8, indicating that this system can adjust the OH^−^ ions concentration in aqueous electrolyte. Besides, XRD patterns of Na_0.44_MnO_2_ in dual-ion cell during cycling are characterized as displayed in Fig. S9. The diffraction peaks of Na_0.44_MnO_2_ show a quasi-regular change, suggesting that the variation of Na_0.44_MnO_2_ during cycling is quasi-reversible. The result is also in good agreement with previous study [44]. Figure 3d presents the cycling performance of this cell. It can retain the capacity of 43.8 mAh g^−1^ after 15 cycles. When cycled to 31 cycles, the dual-ion cell still provides 31.5 mAh g^−1^, showing its potential of application.

## Conclusions

In summary, we propose a novel electrode material Cu_3_(PO_4_)_2_ as an anion container for aqueous dual-ion cell. The sample prepared by a simple precipitation method consists of two phases which are Cu_3_(PO_4_)_2_ and Cu_3_(PO_4_)_2_·3H_2_O. When tested in the three-electrode cell, it can deliver a reversible capacity of 115.6 mAh g^−1^ with a charge plateau of −0.17 V versus Ag/AgCl. Our investigation for the reaction mechanism of Cu_3_(PO_4_)_2_ reveals that the initial capacity loss of this material comes from the decomposition of Cu_3_(PO_4_)_2_ into Cu_2_O, and such transformation is irreversible. Besides, Cu_3_(PO_4_)_2_ reacts with OH^−^ ions instead of PO_4_^3−^ ions after the initial discharge process. Eventually, an available aqueous dual-ion cell has been successfully constructed by applying pretreated Cu_3_(PO_4_)_2_ and Na_0.44_MnO_2_ as anode and cathode. It can provide a discharge capacity of 52.6 mAh g^−1^ with plateaus at 0.70 and 0.45 V, exhibiting its potential of application. On the basis of this work, our next study shall focus on adjustment of the OH^−^ ions concentration in electrolyte by using this dual-ion cell.

## Supplementary Information

Below is the link to the electronic supplementary material.

Supplementary Information1 (pdf 656 kb)
