# Supplementary material for: Cu3(PO4)2: Novel Anion Convertor for Aqueous Dual-Ion Battery
Source: Nanomicro Lett. 2021 Jan 4;13:41. doi: 10.1007/s40820-020-00576-1 (PMC8187538; doi:10.1007/s40820-020-00576-1)
Supplement: Supplementary file 1 — Supplementary Information1 (pdf 656 kb) [file 40820_2020_576_MOESM1_ESM.pdf]

Supporting Information for

$Cu_3(PO_4)_2$: Novel Anion Convertor for Aqueous Dual-Ion Battery

Haoxiang Yu[1, 2, †], Chenchen Deng[1, †], Huihui Yan[1], Maoting Xia[1], Xikun Zhang[1], Zhen-Bo Wang[2, *], Jie Shu[1, 3, *]

[1]School of Materials Science and Chemical Engineering, Ningbo University, Ningbo, Zhejiang 315211, P. R. China

[2]MIIT Key Laboratory of Critical Materials Technology for New Energy Conversion and Storage, School of Chemistry and Chemical Engineering, State Key Lab of Urban Water Resource and Environment, Harbin Institute of Technology, Harbin, Heilongjiang 150001, P. R. China

[3]Key Laboratory of Advanced Energy Materials Chemistry (Ministry of Education), College of Chemistry, Nankai University, Tianjin 300071, P. R. China

[†]Haoxiang Yu and Chenchen Deng contributed equally to this work

*Corresponding authors. E-mail: shujie@nbu.edu.cn (Jie Shu), wangzhb@hit.edu.cn (Zhen-Bo Wang)

S1 Experimental Section

$Cu_3(PO_4)_2$ was synthesized by a facile precipitation reaction. Stoichiometric amounts of $CuCl_2 \cdot 2H_2O$ and Na_3PO_4 were firstly dissolved in distilled water, respectively. Na_3PO_4 solution was then dropped into $CuCl_2$ solution under continuous stirring and the suspension was formed. After aged for 24 h, the sky blue precipitates were filtered and washed with distilled water until the presence of chloride ions could not be detected in the filtrate.

$Na_{0.44}MnO_2$ was prepared by a simple solid state method. Stoichiometric amounts of Na_2CO_3 (5 mol% excess) and $Mn(CH_3COO)_2 \cdot 4H_2O$ were ball milled at 500 rpm for 12 h and subsequently heated in air at 900 °C for 10 h to yield the product.

The morphology of $Cu_3(PO_4)_2$ was recorded by scanning electron microscopy (SEM; Hitachi SU-70). The X-ray diffraction (XRD) pattern of $Cu_3(PO_4)_2$ was collected using an X-ray diffractometer (Bruker D8 Advance) equipped with a Cu Kα radiation source. TG analysis was conducted using a NETZSCH STA409 PG/PC instrument.

Three-electrode cells were used to assess the electrochemical performance of $Cu_3(PO_4)_2$. For the fabrication of the working electrodes, $Cu_3(PO_4)_2$ was blended with acetylene black and polytetrafluoroethylene in the weight ratio of 8:1:1. Subsequently, the mixture was pressed into sheets and pasted onto carbon papers for testing. The counter and reference electrodes were platinum foil and Ag/AgCl (3 M KCl), respectively. The electrolyte was 0.75 M Na_2HPO_4 aqueous solution. All the

electrochemical characterizations of $Cu_3(PO_4)_2$ were conducted on a LANHE CT2001A battery test system.

S2 Supplementary Figures

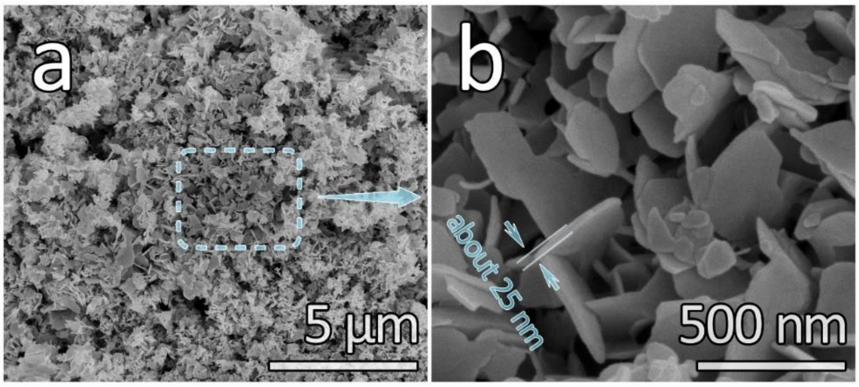

Fig. S1 SEM images of $Cu_3(PO_4)_2$

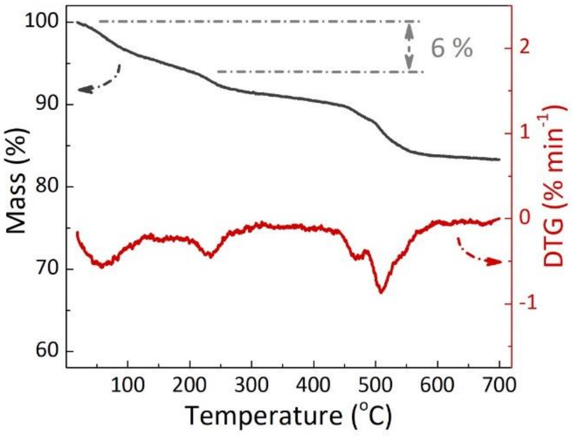

Fig. S2 TG curve of $Cu_3(PO_4)_2$ powder

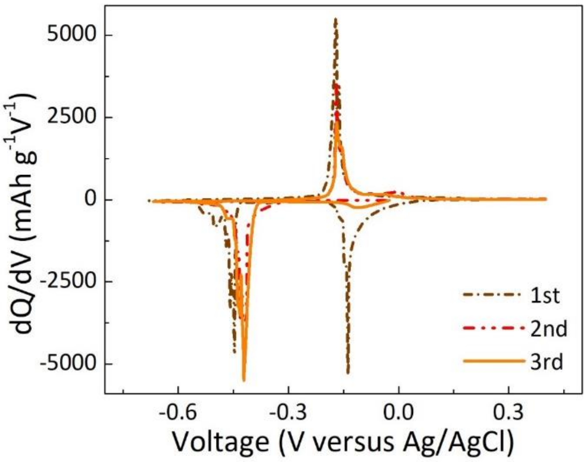

Fig. S3 Differential dQ/dV plots of $Cu_3(PO_4)_2$ between -0.7 and 0.4 V versus Ag/AgCl in the initial three cycles

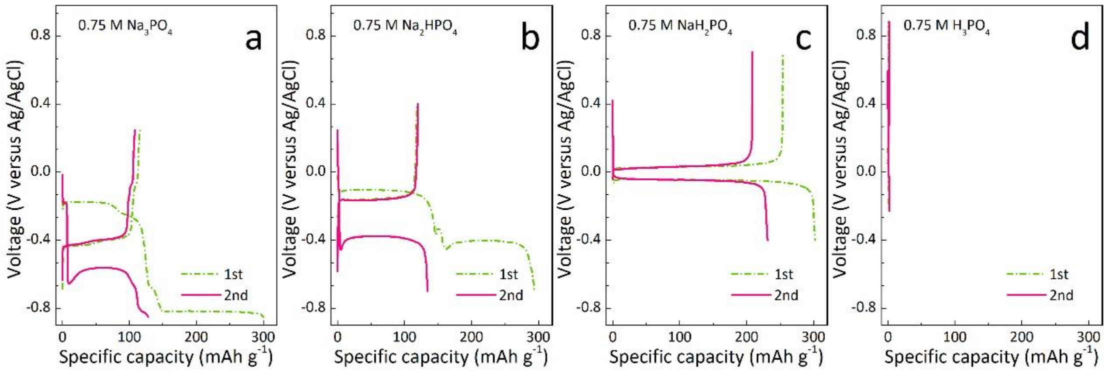

Fig. S4 Galvanostatic discharge/charge profiles of $Cu_3(PO_4)_2$ electrode at 50 mA g^{-1} in electrolytes with different concentration of OH^- ions: **a** 0.75 M Na_3PO_4; **b** 0.75 M Na_2HPO_4; **c** 0.75 M NaH_2PO_4; **d** 0.75 M H_3PO_4

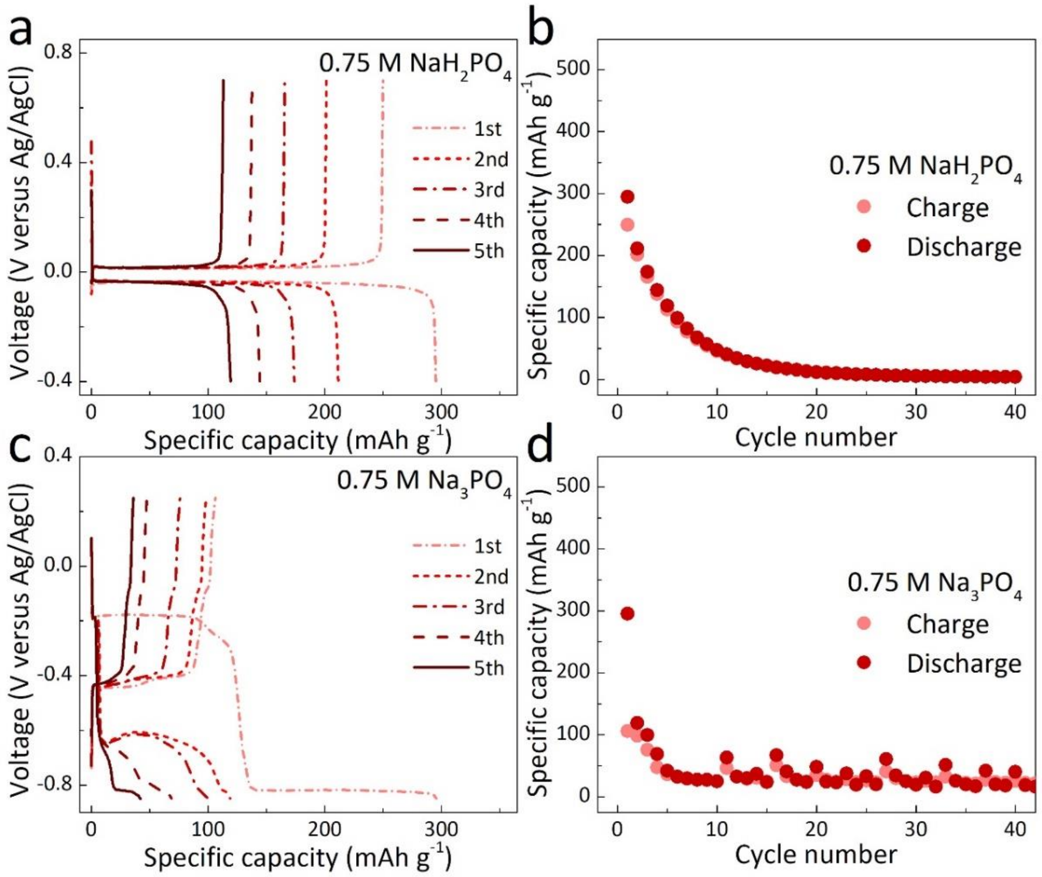

Fig. S5 Galvanostatic discharge/charge profiles and the corresponding cycling performances of $Cu_3(PO_4)_2$ electrode at 50 mA g^{-1} in electrolytes with different concentration of OH^- ions: **a**, **b** 0.75 M NaH_2PO_4; **c**, **d** 0.75 M Na_3PO_4

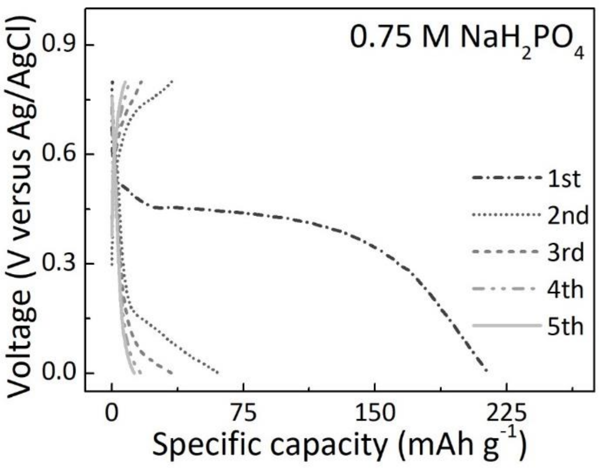

Fig. S6 Galvanostatic discharge/charge profiles of $Na_{0.44}MnO_2$ in 0.75 M NaH_2PO_4 aqueous solution

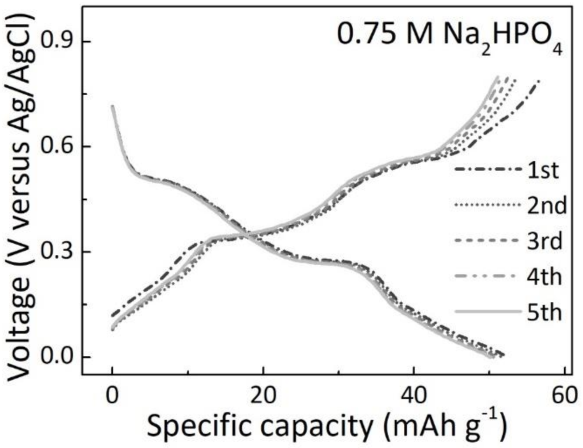

Fig. S7 Galvanostatic discharge/charge profiles of $Na_{0.44}MnO_2$ in 0.75 M Na_2HPO_4 aqueous solution

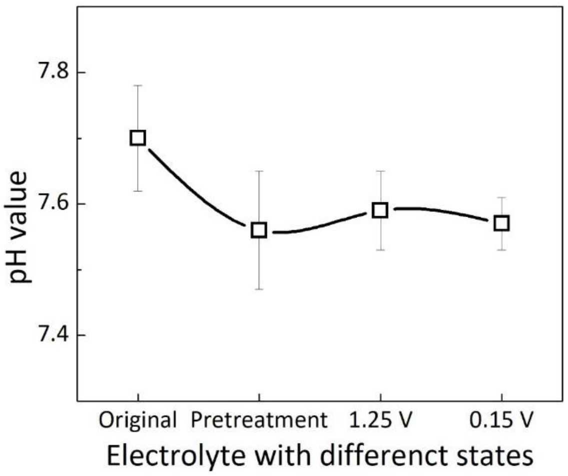

Fig. S8 Variation for pH value of electrolyte during cycling in pretreated $Cu_3(PO_4)_2/Na_{0.44}MnO_2$ dual-ion cell

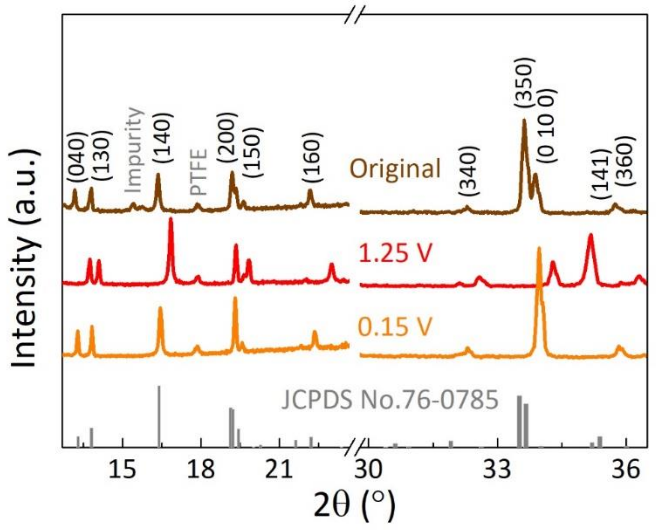

Fig. S9 XRD patterns of $Na_{0.44}MnO_2$ electrode in pretreated $Cu_3(PO_4)_2/Na_{0.44}MnO_2$ dual-ion cell during cycling

According to previous references [36, 44], $Na_{0.44}MnO_2$ is isostructural with $Na_4Mn_4Ti_5O_{18}$. Therefore, we use $Na_4Mn_4Ti_5O_{18}$ to match with $Na_{0.44}MnO_2$.

Table S1 Comparison for operating voltage of pretreated $Cu_3(PO_4)_2/Na_{0.44}MnO_2$ dual-ion cell in this work with other cells in the literatures

**Cell**	**Operating voltage (V)**	**References**
$Na_{0.44}MnO_2$//pretreated $Cu_3(PO_4)_2$	0.70 & 0.45	This work
K20//TiS_2-10CNT	~0.4	[38]
MnO_2//Ag	~0.4	[40]
S//Fe	~0.36	[41]
$Na_{0.44}MnO_2$//BiOCl	0.35	[39]
$Na_2Mn_5O_{10}$/AgCl	0.3	[23]
$FePO_4$//Ag	~0.2	[40]
$FePO_4$//Ag	~0.2	[42]
S//Cu	~0.15	[43]
